# Depressive symptoms can negatively influence patient reported disease severity after subthalamic nucleus stimulation for Parkinson's disease

**DOI:** 10.1177/1877718X251354933

**Published:** 2025-06-26

**Authors:** Christine Girges, Alexis de Roquemaurel, Nirosen Vijiaratnam, Jennifer Foley, Joseph Candelario, Maricel Salazar, Catherine Milabo, John Esperida, Tim Grover, Harith Akram, Jonathan Hyam, Marie T Krüger, Ludvic Zrinzo, Patricia Limousin, Thomas Foltynie

**Affiliations:** 1 Department of Clinical and Movement Neurosciences, University College London, London, UK; 2 UCL Functional Neurosurgery Unit, National Hospital for Neurology and Neurosurgery, London, UK; 3 Institute of Neurological Sciences, Queen Elizabeth University Hospital, Glasgow, UK; 4 Clinical Research Centre for Movement Disorders and Gait, Kingston Centre, Parkinson's Foundation Centre of Excellence, Monash Health, Cheltenham, Victoria, Australia; 5 School of Clinical Sciences, Department of Medicine, Monash University, Clayton, Victoria, Australia; 6 Department of Neuropsychology, National Hospital for Neurology and Neurosurgery, London, UK; 7 Department of Stereotactic and Functional Neurosurgery, University Medical Centre Freiburg, Faculty of Medicine, University of Freiburg, Freiburg im Breisgau, Germany

**Keywords:** Parkinson's disease, deep brain stimulation, depressive symptoms, outcome measures

## Abstract

**Background:**

Depression can negatively influence an individual's perception of their disease. Although subthalamic nucleus deep brain stimulation (STN-DBS) is an effective treatment for Parkinson's disease (PD), some patients do not appreciate benefits despite showing objective improvements in motor function.

**Objective:**

We explored the impact of depressive symptoms on self-reported outcomes of PD severity in patients who underwent STN-DBS.

**Methods:**

Assessments took place preoperatively and 2-years after surgery. Patients completed the Hospital Anxiety and Depression Scale (HADS), Unified Parkinson's Disease Rating Scale (UPDRS) Parts 2 and 4, Gait and Falls Questionnaire, Parkinson's Disease Questionnaire-39 (PDQ-39), and the Non-motor Symptoms Scale. The UPDRS Part 3 (motor examination) was also performed. Patients were dichotomized into two groups (normal or high) based on their postoperative follow-up HADS depression score.

**Results:**

Eighteen patients (33.3%) were assigned to the high group (hHADS-D), and 36 patients (66.7%) were assigned to the normal group (nHADS-D). The UPDRS Part 3 OFF-medication score improved to a similar extent in both groups, and participants experienced a similar reduction in their levodopa equivalent daily dose following STN-DBS. Unlike the nHADS-D group, however, hHADS-D patients did not self-report improvements on any clinical outcome measure at follow-up from baseline, and instead indicated a significant worsening on the UPDRS Part 2 ON-medication and PDQ-39 cognition domain. This was not explicable by their preoperative non-motor symptom burden, nor changes in dopaminergic medications. There were no differences between groups in terms of proportion using anti-depressants, surgical complications or postoperative side effects.

**Conclusions:**

Depressive symptoms may play a significant role in subjective self-reporting, and should be carefully considered when evaluating STN-DBS effectiveness and managing patients postoperatively.

## Plain language summary

Feeling low can affect our outlook, meaning we may pay less attention to positive events and interpret certain situations more pessimistically. Deep brain stimulation (DBS) is a surgical treatment for Parkinson's disease (PD), and helps to improve motor symptoms (e.g., tremor, stiffness and slowness) and consequently quality of life. Some people with PD, however, do not feel that this treatment has helped them. We therefore explored the impact of depressive symptoms on self-reported questionnaires, measuring the patients’ own perception and experience of their disease two years following DBS surgery. Fifty-four people with PD were divided into two groups (normal or high burden of depression) based on a score from a self-reported questionnaire which primarily assessed anhedonia (loss of pleasure). We found the groups to be comparable in terms of how long they experienced PD for and how severe the motor symptoms were when assessed by a doctor. The extent of their motor symptom improvement and dopaminergic medication reduction were also similar after surgery, as were the number of side effects experienced by each group and the number of adjustments needed to optimize the treatment. However, the high depression group did not report improvements in their quality of life, walking and balance, and non-motor symptoms (such as memory, sleep and constipation). Instead, they reported a significant worsening in their cognition and how PD impacted on their daily activities when medicated. These results thus suggest that depressive symptoms can negatively influence how a patient may judge their symptoms, and reinforces that it is important to identify and treat any low mood to improve outcomes following DBS.

## Introduction

Subthalamic nucleus deep brain stimulation (STN-DBS) is a highly effective treatment for Parkinson's disease (PD), usually leading to significant improvements in motor symptoms and health-related quality of life (HR-QoL) in well selected candidates.^1^ Despite this, up to a quarter of patients perceive their overall outcome as disappointing, even in the absence of surgical complications or device issues.^2,3^ The clinician may struggle to effectively optimize stimulation parameters in such cases, which might (in part) relate to a disconnect between objective and subjective evaluations of STN-DBS effectiveness.

A low or depressed mood can foster congruent cognitive biases and may explain why some patients do not appreciate a subjective benefit from STN-DBS.^4,5^ It can be difficult to disentangle whether the cause of depression is primarily a reactive response to disability or changes in dopaminergic medication, or if it is a non-motor manifestation of the neurodegenerative process itself. Irrespective of the possible contributory causes, a depressed mood could be a major bias for research outcomes, as well as influencing the patient's perception of their own clinical outcome.^2,6^

Several studies have reported both positive and negative effects of STN-DBS on mood, and associate such changes with stimulation parameters, coordinates of the electrode contacts, and reductions in dopaminergic medications.^7,8^ The aim of this work, however, was to simply explore the impact of depressive symptoms on self-reports of PD severity and HR-QoL in a cohort of patients two years after STN-DBS surgery. From an ethical and clinical standpoint, identifying people who may be at a higher risk of subjectively perceiving their outcome as unsatisfactory is important and could inform postoperative management.^2,3^

## Methods

### Population

Patient selection for STN-DBS, preoperative assessments and neurosurgical procedures have been described previously.^9^ A sub-group of patients who were treated with bilateral STN-DBS between January 2014 and October 2018 at our institution were enrolled in this study. To be included, patients must have completed the Hospital Anxiety and Depression Scale (HADS) prior to surgery (baseline) and 2-years after surgery (postoperative follow-up). Follow-up appointments were performed as part of standard care to review outcomes of DBS. This work was carried out in accordance with the Declaration of Helsinki and written informed consent was taken for data to be used in medical research.

### Clinical outcomes

Clinical assessments took place at baseline and 2-years after surgery. Patients completed the following self-reported questionnaires: (1) the HADS measured anxiety (HADS-A) and depressive (HADS-D) symptoms, which were scored separately; (2) the Parkinson's Disease Questionnaire (PDQ-39) measured HR-QoL across eight dimensions. Each dimension total score ranged from 0 to 100, and an overall summary index (SI) was computed by taking a weighted average of these scores; (3) the Non-motor Symptom Scale (NMSS) measured the severity and frequency of non-motor symptoms (the mood/cognition domain was removed from the total score as it would correlate highly with the HADS-D); and (4) the Gait and Falls Questionnaire (GFQ) measured gait and balance. Higher scores on these scales represented more severe symptoms.

Cognition was assessed with the Mini Mental State Examination (MMSE) at baseline, and Montreal Cognitive Assessment (MoCA) at follow-up. For comparative purposes, we converted the MoCA to MMSE according to published methods.^10^

Evaluation of overall clinical severity was based on the Unified Parkinson's Disease Rating Scale (UPDRS). The motor examination (Part 3) was performed OFF and ON-medication at both time-points, with the addition of ON-stimulation at follow-up. Part 2 (patient-reported experiences of daily living OFF and ON-medication) and Part 4 (motor complications) were completed. As UPDRS Part 1 assesses cognitive and neuropsychiatric symptoms and therefore overlaps with depression ratings, it was not included in the current analysis.

### Statistical analysis

Patients were dichotomized into two groups based on their 2-year follow-up HADS-D score. A normal or high burden of depressive symptoms was defined as a HADS-D score of 0–7 or ≥ 8, respectively.^11^

For continuous variables, independent samples t-tests were applied to compare the normal and high depression groups at two separate timepoints (baseline and follow-up). Paired samples t-tests were used to compare differences between baseline and follow-up within each group. Chi-squared tests were used for categorical data.

Univariable linear regression was used to further evaluate the impact of postoperative depressive symptoms on self-reported outcome measures across the entire cohort. We chose to specifically investigate the UPDRS Part 2 ON-medication, PDQ-39SI and PDQ-39 cognition domain as these outcomes were shown to worsen postoperatively in the hHADS-D group. The predictor variables were: (1) HADS-D scores at follow-up; (2) age; (3) gender with ‘male’ as the reference category; (4) disease duration; (5) objective clinical severity (UPDRS Part 3 ON-medication ON-stimulation); (5) anti-depressant use with ‘no use’ as the reference category; (6) dopamine agonist reduction; and (7) preoperative scores that were considerably different between groups (HADS-D, NMSS, GFQ, PDQ-39SI). Multivariable linear regression models were then constructed, adjusting for potential confounds only if they were significant predictors in the univariable models. A Bonferroni correction was applied to adjust for the number of statistical tests conducted on each variable. Significance was therefore set to p < 0.017). Analyses were conducted in SPSS (Version 28).

Mean (SD) anti-depressant daily doses (fluoxetine equivalent) were calculated using the method proposed by Hayasaka and colleagues.^12^

## Results

Fifty-four patients (38 males, mean age = 60.04 years, SD = 8.49, range = 41–75 years) were reviewed 2.33 years (SD = 0.95) after STN-DBS surgery. Eighteen patients (33.3%) scored ≥ 8 on the HADS-D at the postoperative follow-up and were assigned to the high group (hHADS-D), while the remaining 36 patients (66.7%) were assigned to the normal group (nHADS-D).

### Factors that influence depressive symptoms at follow-up

#### Baseline characteristics

Compared to the nHADS-D group, the hHADS-D group reported a higher level of anxious (HADS-A: t _(49)_ = -2.46, p = 0.017) and depressive (HADS-D: t _(49)_ = -2.40, p = 0.020) symptoms prior to surgery, although the latter result did not remain significant after correcting for multiple comparisons. There were no significant baseline differences between groups in terms of demographic characteristics, proportion using anti-depressants, or overall clinical severity as measured by the UPDRS Parts 2 to 4 (Supplemental Table 1). The hHADS-D group exhibited more impaired scores on the PDQ-39SI (t _(50)_ = -2.69, p = 0.010), GFQ (t _(49)_ = -2.68, p = 0.010) and NMSS (t _(48)_ = -2.08, p = 0.043 uncorrected). The mean anti-depressant daily dose (fluoxetine equivalent) prescribed was higher in the hHADS-D group (31.26 mg SD = 25.72) compared to the nHADS-D group (15.07 mg SD = 8.71), but not significantly so.

#### Cross-over between groups from baseline to follow-up

**Figure 1.**
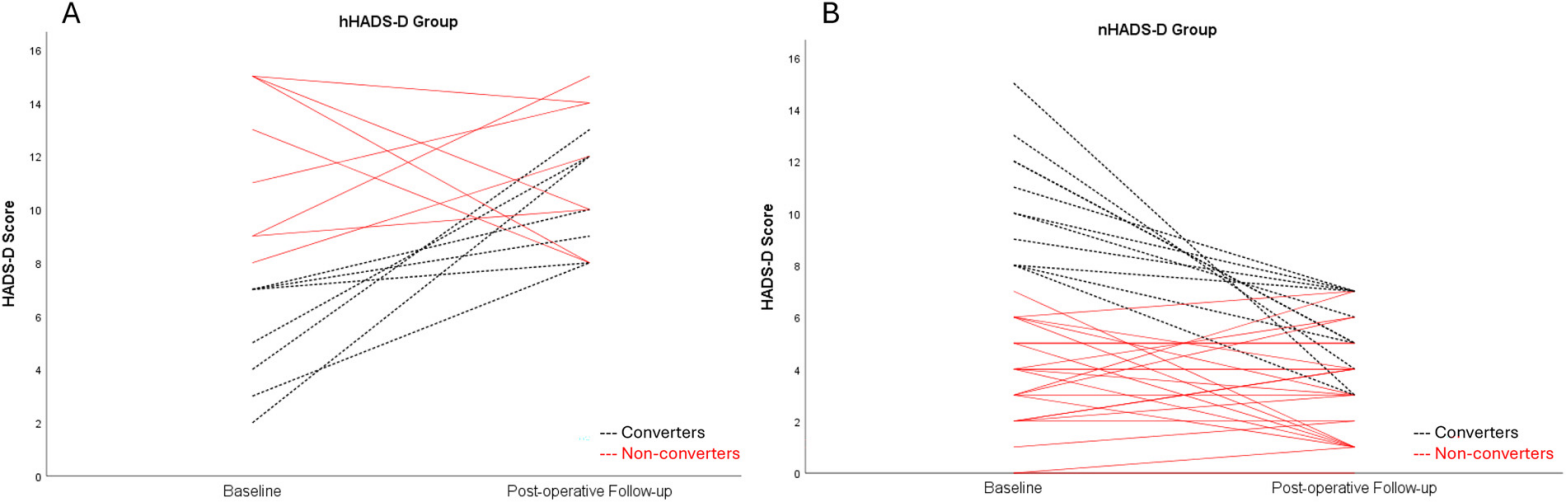
Panels A and B illustrate the individual trajectories for each patient in the hHADS-D and nHADS-D groups, respectively. The black dotted lines indicate patients who converted from one group to another between baseline and the postoperative follow-up.

In the nHADS-D group, 11 patients (30.6%) scored ≥ 8 on the HADS-D at baseline and converted to a normal score at follow-up. The remaining 25 patients (69.4%) scored normally at both time-points. In the hHADS-D group, 7 patients (38.9%) scored normally on the HADS-D at baseline and converted to a high score at follow-up, while 8 patients (44.4%) scored ≥ 8 at both time-points (Figure 1). A baseline HADS-D score was missing for 3 patients in this group (16.7%). Baseline HADS-D scores significantly predicted postoperative HADS-D scores (R^2^ = 0.16, F _(1,49)_ = 9.64, coefficient 0.38, 95% CI 0.13 to 0.62, p = 0.003).

#### Objective clinical severity and levodopa equivalent daily dose (LEDD) at follow-up

UPDRS Part 3 significantly improved OFF-medication in the nHADS-D (−34.9%; t _(34)_ = 7.63, p < 0.001) and hHADS-D patients (−39.0%; t _(17)_ = 3.79, p = 0.001), with no difference in the magnitude of change from baseline between groups (p = 0.447). Total LEDD was also markedly reduced in both groups (nHADS-D: −37.9%; t _(35)_ = 6.08, p < 0.001; hHADS-D: −47.4%; t_(17)_ = 6.91, p < 0.001) and to a similar extent (p = 0.121). Dopamine agonists were reduced to a higher extent in the hHADS-D group (−80.7%) compared to the nHADS-D group (−58.9%), but the difference was not significant (p = 0.478).

#### Self-reported measures of neuropsychiatric state and clinical severity at follow-up

The nHADS-D group reported a significant improvement from their baseline HADS-D (−31.8%; t _(35)_ = 3.15, p = 0.003) and HADS-A scores (−23.5%; t _(35)_ = 2.50, p = 0.017). In contrast, the hHADS-D group reported a slight worsening of their neuropsychiatric state which did not reach statistical significance (Table 1). The magnitude of change was significantly different between the groups for the HADS-D (t _(49)_ = -3.28, p = 0.002) but not HADS-A (p = 0.090).

**Table 1.** Baseline and postoperative scores of the normal and high depression symptom groups at follow-up (2.33 years after STN-DBS surgery).

	Normal Group (n = 36)			High Group (n = 18)		
	Baseline	Follow-up	*p*	Baseline	Follow-up	*p*
Total LEDD, mg	1094.89 (550.09)	680.31 (405.34)	**<0**.**001**	1255.78 (398.64)	661.11 (484.90)	**<0**.**001**
Dopamine Agonist LEDD, mg	193.61 (272.36)	79.64 (88.93)	**0**.**017**	216.94 (361.90)	41.94 (65.6)	0.044
HADS-D	5.75 (3.79)	3.92 (2.10)	**0**.**003**	8.67 (4.35)	10.87 (2.45)	0.114
HADS-A	6.50 (4.14)	4.97 (3.64)	**0**.**017**	9.40 (2.92)	9.87 (2.88)	0.655
UPDRS Part 2 OFF-med	20.06 (7.64)	16.46 (7.64)	0.025	21.75 (10.95)	22.63 (8.90)	0.723
UPDRS Part 2 ON-med	7.17 (5.73)	7.74 (4.22)	0.627	8.53 (8.62)	16.27 (8.80)	**0**.**001**
UPDRS Part 3 OFF-med	41.54 (12.63)	27.06 (11.39)*	**<0**.**001**	47.56 (15.98)	29.00 (8.35)*	**0**.**001**
UPDRS Part 3 ON-med	16.24 (6.48)	17.33 (6.83)*	0.363	17.41 (8.72)	21.65 (8.17)*	0.181
UPDRS Part 4	8.09 (4.62)	5.40 (2.81)	**0**.**001**	7.00 (4.15)	7.38 (3.10)	0.752
NMSS	60.57 (50.87)	48.51 (33.75)	0.049	91.21 (47.61)	93.57 (42.85)	0.891
NMSS (Minus Domain 3)	49.69 (37.90)	40.63 (25.28)	0.060	75.21 (41.48)	75.21 (33.17)	1.000
GFQ	20.42 (13.15)	11.33 (6.45)	**<0**.**001**	29.87 (12.69)	24.07 (11.60)	0.197
PDQ-39						
*Mobility*	48.96 (25.84)	33.95 (26.63)	**0**.**003**	60.78 (23.99)	63.91 (20.55)	0.620
*ADL*	42.11 (19.73)	27.08 (20.18)	**<0**.**001**	58.33 (20.07)	56.77 (17.53)	0.806
*Emotional Well-being*	24.19 (21.67)	19.91 (17.59)	0.153	36.99 (26.07)	47.13 (18.23)	0.088
*Stigma*	32.32 (27.48)	17.56 (19.48)	**0**.**002**	43.39 (23.65)	44.94 (21.44)	0.829
*Social Support*	18.51 (19.33)	14.81 (16.68)	0.261	28.11 (17.46)	36.46 (22.94)	0.203
*Cognition*	24.68 (21.45)	25.02 (19.53)	0.910	28.93 (14.59)	48.06 (21.62)	**0**.**007**
*Communication*	31.48 (19.13)	34.26 (23.89)	0.392	55.74 (42.69)	63.02 (20.86)	0.571
*Bodily Discomfort*	47.46 (24.79)	31.94 (21.50)	**<0**.**001**	60.42 (21.84)	52.08 (21.40)	0.228
*Summary Index*	33.71 (16.16)	25.56 (13.98)	**0**.**002**	46.58 (15.41)	51.54 (12.09)	0.280
MMSE**	28.71 (1.70)	28.38 (1.77)	0.125	28.63 (1.26)	27.83 (2.04)	0.105

*UPDRS Part 3 at follow-up was always performed ON-stimulation. **MMSE was performed at baseline, and MoCA at follow-up. For comparative purposes, we converted MoCA to MMSE according to published methods. ADL: Activities of daily living.

The nHADS-D group reported a significant improvement compared to baseline on the UPDRS Part 4 (−33.3%; t _(34)_ = 3.59, p = 0.001), GFQ (−44.5%; t _(32)_ = 4.36, p < 0.001) and PDQ-39SI (−24.2%; t _(35)_ = 3.39, p = 0.002; Table 1 reports individual PDQ-39 dimensions). Their experiences of daily living in the OFF-medication state (as measured by the UPDRS Part 2) also improved following STN-DBS (−17.9%). However, this effect did not survive Bonferroni correction (p = 0.025). The hHADS-D group, conversely, reported a significant worsening on the UPDRS Part 2 ON-medication (47.6%; t _(14)_ = 4.37, p = 0.001) and PDQ-39 cognition domain (39.8%; t _(15)_ = -3.15, p = 0.007). There were no other significant differences between baseline and follow-up for the hHADS-D group. Supplemental Table 2 further demonstrates that the hHADS-D group consistently reported more severe symptoms compared to the nHADS-D group at follow-up, despite having similar LEDDs, objective clinical severity scores and anti-depressant use. The magnitude of change from baseline to follow-up was significantly different between nHADS-D and hHADS-D patients for the UPDRS Part 2 ON-medication (t _(48)_ = -3.37, p = 0.001) and PDQ-39SI (t _(50)_ = -2.82, p = 0.007) scores only.

#### Anti-depressant use at follow-up

Ongoing treatment with anti-depressants was observed in 6 of 18 (33.3%) hHADS-D patients and in 8 of 36 (22.2%) nHADS-D patients at follow-up. The mean anti-depressant daily dose (fluoxetine equivalent) prescribed was 15.99 mg in the nHADS-D group and 27.58 mg in the hHADS-D group, which remained largely unchanged from baseline. Compared to the treated nHADS-D patients, the treated hHADS-D patients scored higher on several self-reported questionnaires including the UPDRS Part 2, NMSS, GFQ and PDQ-39 (descriptive data are presented in Table 2 due to the small sample size of this subgroup).

**Table 2.** Clinical characteristics of the patients who were receiving anti-depressant treatment at follow-up (2.33 years after STN-DBS surgery).

	Mean (SD)	
	nHADS-D (n = 8)	hHADS-D (n = 6)
Total LEDD, mg	863.25 (353.89)	992.67 (608.59)
Dopamine Agonist LEDD, mg	143.38 (112.06)	55.00 (89.83)
HADS-D	4.00 (1.93)	9.00 (1.55)
HADS-A	4.75 (3.77)	11.17 (2.56)
UPDRS Part 2 OFF-med	15.63 (8.25)	23.50 (9.48)
UPDRS Part 2 ON-med	6.13 (3.44)	15.33 (9.25)
UPDRS Part 3 OFF-med ON-stim	25.63 (13.16)	26.67 (7.37)
UPDRS Part 3 ON-med ON-stim	15.14 (2.97)	18.67 (7.84)
UPDRS Part 4	6.63 (2.97)	6.67 (3.01)
NMSS (Minus Domain 3)	39.75 (25.20)	57.67 (36.20)
GFQ	10.14 (7.82)	32.17 (5.74)
PDQ-39SI	24.06 (14.60)	45.42 (20.69)

### Association between depressive symptoms and negative self-reporting of disease severity at follow-up

Results from the univariable linear regression models are presented in Table 3. The follow-up UPDRS Part 2 (ON-medication) was significantly predicted by the follow-up HADS-D, UPDRS Part 3 (ON-medication ON-stimulation) and baseline GFQ scores. The PDQ-39 was influenced by the same variables, accompanied by a non-significant trend association with the baseline NMSS scores.

**Table 3.** Univariable linear regression models were performed to explore predictors of UPDRS part 2 (on-medication), PDQ-39SI and PDQ-39 cognition domain scores at the postoperative follow-up (2.33 years after STN-DBS surgery).

Outcome measure	Coefficients and CI	p
*UPDRS Part 2 ON-med*		
Follow-up HADS-D	0.97 (0.53 to 1.41)	**<0**.**001**
Gender	−1.20 (−5.39 to 2.99)	0.567
Age	0.05 (−0.17 to 0.28)	0.644
DD at Follow-up	0.36 (−0.07 to 0.80)	0.098
UPDRS Part 3 OFF-med ON-stim	0.39 (0.14 to 0.63)	**0**.**003**
Anti-depressant Use	−0.33 (−4.62 to 3.95)	0.876
Baseline HADS-D	0.13 (−0.37 to 0.62)	0.611
Baseline NMSS	−0.01 (−0.05 to 0.03)	0.687
Baseline NMSS (Minus Domain 3)	−0.01 (−0.06 to 0.04)	0.713
Baseline GFQ	0.18 (0.03 to 0.33)	**0**.**017**
Baseline PDQ-39SI	0.09 (−0.03 to 0.21)	0.135
Dopamine Agonist Reduction	0.001 (−0.01 to 0.01)	0.723
*PDQ-39SI*		
Follow-up HADS-D	2.84 (1.85 to 3.83)	**<0**.**001**
Gender	−0.36 (−11.11 to 10.39)	0.947
Age	−0.12 (−0.71 to 0.46)	0.670
DD at Follow-up	1.15 (0.03 to 2.26)	0.045
UPDRS Part 3 OFF-med ON-stim	0.92 (0.33 to 1.51)	**0**.**003**
Anti-depressant Use	0.06 (−11.14 to 11.27)	0.991
Baseline HADS-D	1.31 (0.11 to 2.51)	0.034
Baseline NMSS	0.11 (0.02 to 0.21)	0.025
Baseline NMSS (Minus Domain 3)	0.17 (0.02 to 0.27)	0.024
Baseline GFQ	0.60 (0.26 to 0.94)	**<0**.**001**
Baseline PDQ-39SI	0.59 (0.34 to 0.84)	**<0**.**001**
Dopamine Agonist Reduction	0.01 (−0.01 to 0.02)	0.417
*PDQ-39 Cognition*		
Follow-up HADS-D	2.66 (1.26 to 4.07)	**<0**.**001**
Gender	−9.65 (−22.86 to 3.57)	0.149
Age	0.15 (−0.59 to 0.88)	0.690
DD at Follow-up	1.48 (0.08 to 2.87)	0.039
UPDRS Part 3 OFF-med ON-stim	0.36 (−0.49 to 1.20)	0.397
Anti-depressant Use	−4.26 (−18.25 to 9.74)	0.545
Baseline HADS-D	1.34 (−0.18 to 2.85)	0.082
Baseline NMSS	0.13 (0.01 to 0.25)	0.040
Baseline NMSS (Minus Domain 3)	0.16 (0.002 to 0.32)	0.047
Baseline GFQ	0.31 (−0.17 to 0.79)	0.198
Baseline PDQ-39SI	0.57 (0.22 to 0.92)	**0**.**002**
Dopamine Agonist Reduction	0.004 (−0.02 to 0.03)	0.736

DD: disease duration.

In the multivariable comparisons (Table 4), including adjustment for objective clinical severity (UPDRS Part 3 ON-medication ON-stimulation) and/or preoperative factors, the HADS-D at follow-up remained the only significant predictor of the UPDRS Part 2 ON-medication (R^2^ = 0.43, F _(3, 42)_ = 10.40, coefficient 0.89, 95% CI 0.41 to 1.37, p < 0.001), PDQ-39SI (R^2^ = 0.51, F _(4, 42)_ = 11.07, coefficient 2.01, 95% CI 0.91 to 3.11, p < 0.001) and PDQ-39 cognition domain (R^2^ = 0.28, F _(2, 49)_ = 9.63, coefficient 2.04, 95% CI 0.49 to 3.59, p < 0.011).

**Table 4.** Multivariate linear regression.

Outcome measure	Coefficients and CI	p
*UPDRS Part 2 ON-med*		
HADS-D	0.89 (0.41 to 1.37)	**<0**.**001**
UPDRS Part 3 ON-med ON-stim	0.27 (0.04 to 0.50)	0.025
Baseline GFQ	0.07 (−0.06 to 0.20)	0.291
*PDQ-39SI*		
HADS-D	2.01 (0.91 to 3.11)	**<0**.**001**
UPDRS Part 3 ON-med ON-stim	0.54 (0.02 to 1.06)	0.043
Baseline GFQ	0.07 (−0.29 to 0.42)	0.704
Baseline PDQ-39SI	0.29 (0.01 to 0.58)	0.046
*PDQ-39 Cognition Domain*		
HADS-D	2.04 (0.49 to 3.59)	**0**.**011**
Baseline PDQ-39SI	0.37 (0.004 to 0.73)	0.048

### Surgical complications and postoperative side effects

Three patients (two from the hHADS-D group) suffered postoperative infections of the implantable pulse generator or head wound requiring surgical debridement and antibiotics. One hHADS-D patient experienced prominence of the cables in the neck, corrected with re-tunnelling surgery. Transient postoperative psychosis was noted in one nHADS-D patient. The proportion of side effects reported during follow-up were similarly distributed across the nHADS-D and hHADS-D groups (Table 5), and included speech (17 of 54; 31.5%), gait and balance (18 of 54; 33.3%) and psychiatric (10 of 54; 18.5%) disturbances. The number of adjustments made to stimulation parameters in 2-years did not significantly differ between the nHADS-D and hHADS-D groups (7 versus 9 adjustments, respectively), p = 0.160. The mean amplitude across hemispheres was also comparable between groups at follow-up (nHADS-D: 2.99 mA, SD = 1.35; hHADS-D: 3.16 mA, SD = 1.79), p = 0.709.

**Table 5.** Proportion of patients who reported side effects with STN-DBS in the follow-up period (2.33 years after surgery).

Side effects (%)	Total cohort	nHADS-D	hHADS-D
Speech	17/54 (31.5)	7/17 (41.2)	10/17 (58.8)
Gait and Balance	18/54 (33.3)	8/18 (44.4)	10/18 (55.6)
Psychiatric	10/54 (18.5%)	5/10 (50.0)	5/10 (50.0)

## Discussion

We have shown that higher levels of depressive symptoms can adversely influence a patient's reported severity of their disease following STN-DBS. Unlike the nHADS-D group, the hHADS-D group did not report improvements on any clinical outcome measure at follow-up from baseline, and instead indicated a significant worsening on the UPDRS Part 2 ON-medication and PDQ-39 cognition domain. The PDQ-39 communication score was also worse at follow-up for hHADS-D patients, potentially reflecting stimulation-induced dysarthria. The number of side effects reported however, and the number of adjustments made to stimulation parameters were comparable between groups. All patients similarly experienced a 30–40% improvement in their motor function OFF-medication (measured objectively by the UPDRS Part 3) at 2-years, suggesting that negative self-reporting of disease severity was not entirely a reactive response to poor motor outcomes or DBS-related adverse events. While the UPDRS Part 3 was unsurprisingly associated with UPDRS Part 2 and PDQ-39SI at follow-up, the postoperative HADS-D scores were a much stronger predictor of these outcomes, even after adjustment for disease severity.

Psychiatric symptoms and cognitive impairment are often associated in PD,^13^ and can negatively impact on disease awareness and insight.^10^ The hHADS-D group indeed self-reported that their cognition was worse at follow-up and additionally showed a slight 1-point decline in their converted MMSE score following STN-DBS compared to the nHADS-D group, although this was not significant and still within the normal range. It is therefore unlikely that the results of this study were unduly influenced by comorbid cognitive impairment. A self-reported decline in cognition could instead potentially be related to unrealistic expectations of DBS.^2^

The nHADS-D group reported a 2-point improvement in their mood postoperatively (−31.8% from baseline). It is not uncommon for STN-DBS to have a positive effect on mood, particularly in those with a longer disease duration and greater functional impairment.^14^ As our groups did not differ in this regard, it is possible that other relevant determinants of mood improvements following STN-DBS may include baseline depression levels, psychosocial dynamics within support networks, having realistic preoperative expectations, and anti-depressant treatment status.^2^ Conversely, the hHADS-D group reported a 2-point worsening (25.4% from baseline) on the HADS-D. Understanding the cause of depressive symptoms in STN-DBS patients postoperatively has been extensively described elsewhere^7,15^ and goes beyond the scope of the current work. It is important to note, however, that both the total LEDD and dopamine agonist LEDD were markedly reduced to a similar extent in both groups at follow-up. The data cannot therefore simply be explained as a result of depressive symptoms developing (or being exacerbated) solely as a result of dopaminergic treatment withdrawal in the hHADS-D group postoperatively, although a delayed onset and individual differences in mesocorticolimbic denervation cannot be completely ruled out.^15^

Stimulation parameters and/or precise electrode location may be related to an increase in depressive symptoms. High-frequency stimulation may inhibit midbrain 5-hydroxytryptamine neurons and evoke depression-related behavioral changes for example,^16^ while more anterior stimulation has been associated with poorer neuropsychological outcomes in those with a psychiatric history.^17^ Electrode placements located more inferiorly and laterally away from the approximated STN also give rise to greater levels of depressive symptoms postoperatively.^18^ We did not find a difference in the mean amplitude between nHADS-D and hHADS-D patients to suggest that an increase in the current delivered to tissues surrounding the electrode contacts was relevant. However, creating volumes of tissue activated for bilateral STN electrodes and modeling the relationship with connectivity maps for each patient would be informative, and future research should explore this within the context of the current findings.

Mood states can evidently influence how a patient perceives and processes information relating to their disease,^19^ thus creating a negative attentional bias when self-reporting. This might be at odds with the neurologist's perspective, who may conclude that the stimulation is appropriately optimized to alleviate the motor manifestations of PD. Patient reported outcomes, however, are becoming an increasingly important adjunct to objective assessments and so we need to ensure that these accounts are not outwardly biased by mood. It is imperative to not only treat the underlying depression adequately, which we know has beneficial effects on motor performance and HR-QoL,^20^ but to also routinely screen for it at the preoperative assessment and postoperative follow-up. This will facilitate both the early detection of depression in undiagnosed cases, plus the review of anti-depressant type/dose in non-responders.^21^

We readily acknowledge that collinearity may exist between the variables studied here, raising the possibility of a bi-directional effect. For example, non-motor symptoms (other than depression) that do not respond to surgery or a poorer HR-QoL could be the driving factor for postoperative depression. Yet, previous research has demonstrated that depression may be the most significant predictor of HR-QoL variability and that the impact of depression exceeds that of motor severity.^22,23^ Randomized controlled trials of paroxetine, nortriptyline and placebo for treatment of PD depression corroborate these findings. Patients who experienced improvements in depression, compared with those who did not, also experienced significant gains in measures of HR-QoL and disability.^20^ Our own findings align with these conclusions, demonstrating that postoperative depressive symptoms are associated with less subjective improvements in HR-QoL, non-motor symptom burden and experiences of daily living, despite significant improvements in motor ability.

An alternative hypothesis is that a combination of preoperative factors such as levodopa refractory symptoms (e.g., axial or non-motor features), patient expectations and pre-existing depression can inform STN-DBS outcomes. The hHADS-D group indeed showed a predisposition for negative self-reporting even at baseline, particularly in the NMSS, GFQ and PDQ-39. They generally experienced more depressive symptoms before surgery too, although this finding did not survive Bonferroni correction. In our regression analyses, we found that only the baseline GFQ significantly predicted postoperative UPDRS Part 2 and PDQ-39SI outcomes. This effect was lost in the multivariable models, again suggesting that postoperative depression is the most strongly associated factor.

Relevant associations between preoperative depression status and a self-reported negative outcome of STN-DBS at 3-months have been previously reported in a group of patients who responded well to surgery.^2^ While major or severe psychiatric disorders are contraindications for DBS, these criteria do not eliminate the sub-group of patients who experience milder subclinical depression or levodopa induced mood swings. As adjuncts to anti-depressant therapy, psychotherapy or counseling might be helpful to stabilize affective states and ensure that postoperative expectations are realistic.^2,3^ Beyond this, a greater understanding of which preoperative non-motor symptoms are most likely to be responsive to stimulation is needed to guide patient expectations and refine patient selection at the pre-assessment stage.

### Limitations and future directions

A limitation of this study is the sole use of the HADS-D to characterize depressive symptoms in patients. While this scale is relatively quick and easy to administer in an outpatient setting, it primarily assesses the emotional aspects of depression (emphasizing anhedonia rather than sadness) and excludes somatic or cognitive symptoms.^24^ More severe depressive symptoms, such as suicidal ideation or vegetative states, are therefore not captured by the HADS-D and could further impact on subjective reports of disease severity following STN-DBS.

For screening purposes, a HADS-D cutoff score of 10/11 has been suggested in PD populations.^24,25^ Because of our small sample size, we opted to use the lower cutoff score of 7/8 (which has been validated in general medical populations, including those with physical illnesses) to ensure more balanced groups.^11,26–28^ We have, however, repeated the analysis with the 10/11 cutoff (data not presented) and the conclusions remain the same. Our regression models also included the HADS-D score as a continuous variable independent of cutoff scores, again indicating a significant association between depressive symptoms and negative self-reporting of disease severity following STN-DBS. We acknowledge that larger scale multi-center studies are needed to further investigate this clinically important question and to exclude the possibility that some associations have occurred by chance given the multiple comparisons conducted and small sample size.

We are also aware that apathy is closely related to depression and is a well-known contributor to self-reported negative outcomes following STN-DBS.^2,3^ Although we did not measure apathy in this cohort, we acknowledge that increased levels may neutralize or mask the beneficial effects of DBS on other outcome measures.^6^

A final limitation is that detailed interim follow-up data at 6 or 12-months was not available, meaning it is difficult to ascertain whether the hHADS-D group experienced initial improvements beyond those objectively noted in motoric ability. HR-QoL, for example, is known to regress to baseline levels after 3-years, and so it may be possible that we have not captured any short-term benefits.^29,30^ This is unlikely, however, given that this was not the case in the nHADS-D group.

In conclusion, we suggest that depressive symptoms may play a significant role in subjective self-reporting, and should be carefully considered when evaluating the success of STN-DBS.
